# Substrate Elasticity Exerts Functional Effects on Primary Microglia

**DOI:** 10.3389/fncel.2020.590500

**Published:** 2020-11-05

**Authors:** Stefan J. Blaschke, Seda Demir, Anna König, Jella-Andrea Abraham, Sabine U. Vay, Monika Rabenstein, Daniel N. Olschewski, Christina Hoffmann, Marco Hoffmann, Nils Hersch, Rudolf Merkel, Bernd Hoffmann, Michael Schroeter, Gereon R. Fink, Maria A. Rueger

**Affiliations:** ^1^Department of Neurology, Faculty of Medicine and University Hospital, The University of Cologne, Cologne, Germany; ^2^Department of Cognitive Neuroscience, Institute of Neuroscience and Medicine (INM-3), Research Centre Jülich, Jülich, Germany; ^3^Department of Mechanobiology, Institute of Biological Information Processing (IBI-2), Research Centre Jülich, Jülich, Germany

**Keywords:** neuroinflammation, microglia, elasticity, polarization, vimentin

## Abstract

Microglia—the brain’s primary immune cells—exert a tightly regulated cascade of pro- and anti-inflammatory effects upon brain pathology, either promoting regeneration or neurodegeneration. Therefore, harnessing microglia emerges as a potential therapeutic concept in neurological research. Recent studies suggest that—besides being affected by chemokines and cytokines—various cell entities in the brain relevantly respond to the mechanical properties of their microenvironment. For example, we lately reported considerable effects of elasticity on neural stem cells, regarding quiescence and differentiation potential. However, the effects of elasticity on microglia remain to be explored.Under the hypothesis that the elasticity of the microenvironment affects key characteristics and functions of microglia, we established an *in vitro* model of primary rat microglia grown in a polydimethylsiloxane (PDMS) elastomer-based cell culture system. This way, we simulated the brain’s physiological elasticity range and compared it to supraphysiological stiffer PDMS controls. We assessed functional parameters of microglia under “resting” conditions, as well as when polarized towards a pro-inflammatory phenotype (M1) by lipopolysaccharide (LPS), or an anti-inflammatory phenotype (M2) by interleukin-4 (IL-4). Microglia viability was unimpaired on soft substrates, but we found various significant effects with a more than two-fold increase in microglia proliferation on soft substrate elasticities mimicking the brain (relative to PDMS controls). Furthermore, soft substrates promoted the expression of the activation marker vimentin in microglia. Moreover, the M2-marker CD206 was upregulated in parallel to an increase in the secretion of Insulin-Like Growth Factor-1 (IGF-1). The upregulation of CD206 was abolished by blockage of stretch-dependent chloride channels. Our data suggest that the cultivation of microglia on substrates of brain-like elasticity promotes a basic anti-inflammatory activation state *via* stretch-dependent chloride channels. The results highlight the significance of the omnipresent but mostly overlooked mechanobiological effects exerted on microglia and contribute to a better understanding of the complex spatial and temporal interactions between microglia, neural stem cells, and glia, in health and disease.

## Introduction

As primary immune cells of the central nervous system (CNS), microglia mediate, and regulate neuroinflammation in health and disease (Ransohoff and El Khoury, 2015). A plethora of stimuli impact microglia function, both under physiological as well as under pathological conditions (Stoll et al., 2002; Hanisch and Kettenmann, 2007). Microglia do not only exert (neuro)-inflammation and cell phagocytosis but also possess supportive functions associated with regeneration such as the induction of synaptic pruning and neuronal plasticity (Paolicelli et al., 2011; Schafer et al., 2012). A tightly controlled regulation of both initiation and termination of microglial activation is vital for healthy development (Lenz and Nelson, 2018) and homeostasis of the CNS (Walberer et al., 2014; Yin et al., 2017). Understanding this complex interplay might expedite microglia-directed therapies in the future (Kim et al., 2015). In parallel to the subdivision of activation states of macrophages in other organs, a pro-inflammatory (M1) and an anti-inflammatory (M2) phenotype in microglia have been proposed upon activation (Tang and Le, 2016) with transitional states in between (Vay et al., 2018). While this concept has been a subject of thorough investigation, more in-depth knowledge of influencing factors, transitional states, or state reversibility is needed to develop novel therapeutic options targeting the microglial response to pathology.

Thus far, most research has addressed the effect of soluble factors like chemokines, cytokines, or other means of cell-cell interaction (Kettenmann et al., 2011; Vay et al., 2018). In contrast, little is known about mechanobiological effects on microglia (Goriely et al., 2015). However, mechanical cues constitute an abundant and regionally as well as temporally diverging factor, the influence of which is often neglected. Tissue elasticity, mostly determined by the properties of the extracellular matrix (ECM), is remarkably different within the brain as one of the softest tissues of the body (Tyler, 2012), but changes, e.g., during aging (Arbogast et al., 1997; Arani et al., 2015), and in the context of brain pathology (Schregel et al., 2012; Streitberger et al., 2012; Chauvet et al., 2016). Glial scarring due to brain injury or neuroinflammation (Wuerfel et al., 2010; Streitberger et al., 2012), cerebral neoplasia (Chauvet et al., 2016) as well as neurodegenerative diseases such as Alzheimer’s (Murphy et al., 2011), are associated with changes in tissue elasticity. The change of viscoelastic properties of the CNS is claimed to be a sensitive biomarker of CNS pathology and, consequently, exploited to assess pathological processes by innovative MRI techniques (MRI elastography; for review see Murphy et al., 2019).

We and others have previously shown that a simulation of mechanical properties on CNS cells *in vitro* is feasible and allows analyzing cell functions under more physiological conditions than provided by regular cell cultures, uncovering essential aspects and mechanobiological properties of neural stem cells and neurons (Pathak et al., 2014; Abraham et al., 2019; Blaschke et al., 2019). Overall, mechanical properties alter the development and behavior not only of mesenchymal stem cells (Murphy et al., 2011), hematopoietic stem cells (Kumar et al., 2013), and cardiomyocytes (Hersch et al., 2013) but also of neural cells like neurons (Abraham et al., 2019), astrocytes (Moshayedi et al., 2014), and neural stem cells (Blaschke et al., 2019). While single reports describe morphological alterations of microglia dependent on underlying substrate elasticity (Moshayedi et al., 2014; Bollmann et al., 2015), the impact of elasticity on microglia at the functional level to date remains elusive.

We previously proposed polydimethylsiloxane (PDMS)-based substrates as a suitable *in vitro* model for the investigation of mechanical influences, given smooth surface topography and excellent biocompatibility (Schellenberg et al., 2014; Abraham et al., 2019; Blaschke et al., 2019). We here hypothesize that the ubiquitous influence of substrate elasticity modulates primary microglia functions and might even alter dynamic microglia reaction upon subsequent stimuli. Accordingly, we here cultivated primary rat microglia on PDMS-based elastomeric substrates of varying stiffness to mimic the biophysical cerebral milieu and analyzed the impact on microglial activation and polarization *in vitro*. Thereby, a more in-depth understanding of the complex regulation of microglia in health and disease states will be gained, allowing better prediction of microglia response and, eventually, novel microglia-based treatment strategies.

## Materials and Methods

### Preparation of Elastomeric Silicone Rubber Substrates

Microglia were seeded on PDMS based substrates with different elastic moduli obtained through variation in the composition of the base (vinyl terminated PDMS) and cross-linkers (methyl-hydro siloxane-dimethylsiloxane) copolymer of a two-component system (Sylgard 184, Dow Corning, Wiesbaden, Germany). Mixing ratios of 10:1, 70:1, and 75:1 (base:cross-linker) were used and elastomers were prepared, as described previously (Hersch et al., 2013). Elastomers were cross-linked at 60°C for 16 h as thick layers in cell culture dishes (Nunclon Multidishes, 4-Well, flat bottom, Thermo Fischer Scientific, Waltham, MA, USA). The calibration of the elasticity of all cross-linked elastomeric mixtures was controlled accurately by indentation, as described before (Ulbricht et al., 2013), to result in elasticities of 1.2 MPa (10:1), 1 kPa (70:1), and 0.6 kPa (75:1).

### Microglia Isolation and Cultivation

Cortices of neonatal Wistar rats of postnatal day one to three were used to obtain primary microglial cell cultures as previously described (Rabenstein et al., 2016). In brief, pups were decapitated, and meningeal layers, as well as blood vessels, were removed. The cortices were incubated in trypsin/EDTA solution (1% trypsin; 0.025% EDTA) for 15 min at 37°C. Afterward, culture medium [Dulbecco’s essential medium (DMEM) with the addition of 10% fetal bovine serum (FBS)], 1% penicillin/streptomycin, and 2 mM L-glutamine was added to stop trypsinization. After removal of trypsin, the cortices were mechanically dissociated, and the cells were resuspended in fresh DMEM and transferred into culture flasks to grow at 37°C with 5% CO_2_ for 10 days, with a change of medium after 3 days. Pure microglial cell subcultures were acquired through detaching microglial cells from the astrocytes co-cultured initially by shaking the culture flasks for 1 h at 250 rpm in an orbital shaker at steadily 37°C. The cells were seeded at a density of 5 × 10^4^ cells per well on various formulations of PDMS-coated plates. As normalization controls, cells were additionally seeded on conventional glass coverslips used for cell culture. At least three independent samples from at least two different preparations were used, with the exact number of replicates individually stated below.

### Stimulation

To analyze the effect of external stimuli, microglia polarization was induced by either LPS or IL-4. Two hours after subcultivation, the cell medium was replaced, and microglia were stimulated with 50 ng/ml LPS or 100 ng/ml IL-4, compared to no stimulation. To subsequently assess microglia memory, cells were treated with IL-4 or no stimulation 24 h before treatment with LPS, as described above.

### Blockage of Stretch-Dependent Cl^−^Channels

Primary microglia were seeded on soft PDMS substrates of 0.6 kPa and cultivated for 24 h. Microglia seeded on conventional glass substrates served as controls. Cells on soft substrates were either treated with 4,4′-diisothiocyano-2,2′-stilbenedisulfonic acid (DIDS), an anion exchange inhibitor previously shown to block stretch-activated Cl^−^ currents (Eder et al., 1998; Hines et al., 2009), or its dissolvent, KHCO_3_ as a negative control for 24 h. Afterward, RNA was extracted for further analyses, as described below.

### Immunocytochemistry

After cell fixation with 4% paraformaldehyde for 10 min, cells were subsequently stained with primary antibodies against iNOS (mouse mAB ab49999 Abcam, Cambridge, UK), against vimentin (mouse mAB ab92547 Abcam, Cambridge, UK), against Iba-1 (rabbit mAB 019-19741, WAKO, Neuss, Germany), and with Hoechst 33342 (Sigma–Aldrich, St. Louis, MO, USA).

Before staining for BrdU, cells were incubated in 2 N HCl for 30 min for antigen retrieval. For visualization, fluorescein-labeled anti-mouse immunoglobin (goat anti-mouse IgG, Alexa Fluor TM 488, Thermo Fisher Scientific, Waltham, MA, USA) and anti-rabbit IgG (goat anti-rabbit IgG, Alexa Fluor TM 568, Thermo Fisher Scientific, Waltham, MA, USA) were used. Cells were counted manually using ten randomly selected pictures per trial (FOV = 730 × 550 μm), taken with a Keyence BZ-9000 inverted fluorescence microscope (Keyence Osaka, Japan). On average, 85 cells per image were evaluated.

Using the ImageJ software (Laboratory of Optical and Computational Instrumentation; Wisconsin, USA), the cell area and roundness of microglial cells, chosen as a criterion of morphology, were measured. Roundness is defined here as area/(πb^2^), where b denotes the longer half axis of the ellipse with equal second central moments as the cell outline.

### Cell Viability Assay

To assess cell viability, dead microglia were stained with propidium iodide (Life Technologies, Darmstadt, Germany) and counterstained, irrespective of viability, with Hoechst 33342 (Sigma–Aldrich GmbH; St. Louis, MO, USA) after 24 or 48 h of cultivation. Fluorescence microscopy was performed as described above. To compare the number of living cells across conditions, a ratio of propidium negative (vital) cells to all cells stained by Hoechst 33342 was assessed. In addition to the live/dead assay, cell death was indirectly assessed measuring lactate dehydrogenase (LDH) release into the media using a colorimetric assay (Pierce LDH assay kit, Thermo Scientific, Waltham, MA, USA). The experiment was performed according to the manufacturer’s protocol. The intensity of the red color formed in the assay was measured at a wavelength of 490 nm (FLUOstar Omega, BMG LABTECH, Ortenberg, Germany), being proportional to LDH activity and thus correlating with the number of damaged cells. Results were normalized to lysed cells as a positive control, while blank culture media served as a negative control.

### BrdU Assay

To assess the ratio of proliferating cells, 10 μM bromodeoxyuridine (BrdU; Sigma–Aldrich GmbH; St. Louis, MO, USA) was added to the cells 6 h before fixation with 4% paraformaldehyde (PFA; Electron Microscopy Sciences; Hatfield, PA, USA). After immunocytochemical staining, fluorescence microscopy was performed as described above. To compare proliferating cells across conditions, a ratio of BrdU positive proliferating cells to all cells stained by Hoechst 33342 was assessed. Ten random images per sample were taken as described above.

### Real-Time Quantitative PCR (RT-qPCR)

RNA was extracted from cultivated cells using the GeneUP total RNA mini Kit (Biotechrabbit; Henningsdorf, Germany), following the manufacturer’s recommendations, while respecting the stimulation protocol. The total RNA concentration and purity were evaluated photometrically. Total RNA was converted to cDNA using the QuantiTect reverse transcription kit (Qiagen; Hilden, Germany) following the manufacturer’s protocol. All primers were obtained from Biolegio (Nijmegen, The Netherlands) and were enlisted, along with the thermal cycler conditions (Table 1). Sample amplification and quantification were executed in the CFX Connect™ Real-Time PCR Detection System (Bio-Rad; Hercules, CA, USA). The integrity of the PCR products was evaluated by melting point analysis and agarose gel electrophoresis. Each sample was normalized to ribosomal protein L13a (RPL13a; ΔCq) as a reference gene and the experimental control condition (ΔΔCq). Mean fold changes were expressed as 2^(−ΔΔCq)^.

**Table 1:** Primer sequences and parameters used for real-time quantitative PCR (RT-qPCR).

	Sequences forward/	Temp. (C°)	Duration (s)
RNA	backward 5′–3′	Step 1/2/3	Step 1/2/3
iNOS	GCTTGTCTCTGGGTCCTCTG/	95/59/72	15/15/45
	CTCACTGGGACAGCACAGAA	
CD206	AACAAGAATGGTGGGCAGTC/	95/56/72	15/15/45
	CCTTTCAGTCCTTTGCAAGC	
Ki67	TCTTGGCACTCACAGTCCAG/	95/58/72	15/15/45
	GCTGGAAGCAAGTGAAGTCC	
Vimentin	GCAGCCTCTATTCCTCGTCC/	95/60/72	15/15/45
	TAGTTGGCGAAGCGGTCATT	
IL-6	CCCAACTTCCAATGCTCTCCT/	95/57.3/72	15/15/45
	AGCACACTAGGTTTGCCGAG	
CD68	GGACACTTCGGGCCATGCTT/	95/60/72	15/15/45
	CCTACAGAGTGGACTGGAGC	
RPL13a	TCCAGGAGCTGTTCAAGCG/		
	CAACACCTTGAGGCGTTCCA		

### Griess Assay

NO release was quantified using a Griess reagent kit (Biotium; Hayward, CA, USA). Accordingly, the supernatant was incubated with Griess reagent, and the NO concentration was evaluated under the manufacturer’s protocol by measuring the optical density (OD) of each sample at 548 nm in a plate reader (FLUOstar Omega: BMG LABTECH; Ortenberg, Germany). Results were normalized to standardized measurements.

### Enzyme-Linked Immunosorbent Assays (ELISA)

The concentrations of pro- (IL-1b) and anti-inflammatory proteins (IGF-1) within the culturing media were measured 24 or 48 h after sub-cultivation. Using the mouse/rat IGF-1 Quantikine ELISA Kit or Rat IL-1β/IL-1F2 Quantikine ELISA Kit (#MG100 or #RLB00; R&D Systems; Minneapolis, MN, Canada), the concentration was measured following the manufacturer’s protocol. OD measurements were performed in the plate reader (FLUOstar Omega: BMG LABTECH; Ortenberg, Germany) at 450 nm wavelength with additional measurements at 540 nm to exclude unspecific signals. Results were normalized to standardized measurements.

### Statistical Analyses

Statistical analyses were performed with IBM SPSS Statistics (Version 25, International Business Machines Corporation IBM, Armonk, NY, USA). Group differences were assessed by one-way analysis of variance (ANOVA) in case of a normal distribution as verified by inspection of data distribution and Shapiro–Wilk test. Additionally, *post hoc* Tukey’s HSD—in case of equal variances across groups—or Games-Howell test in heteroscedasticity were performed to control for multiple comparisons. Otherwise, a Kruskal–Wallis test with *post hoc* Bonferroni correction was conducted. Furthermore, to assess the additional effect of LPS stimulation across the elasticity range, a two-way ANOVA was conducted with elasticity and LPS stimulation as independent variables on vimentin expression in microglia.

Additionally, to further assess within-group differences, simple effects analysis with *post hoc* Bonferroni correction was performed. Statistical significance was assumed at *p* < 5%. Besides the descriptive statistic given by the mean (M) and standard deviations (SD), effect sizes are reported as omega squared (Ω^2^) or the correlation coefficient *r* in case of nonparametric tests. Results were displayed as boxplots showing the median, quartiles, and extreme values, with outliers exceeding the quartile by 1.5-fold of the interquartile range displayed as dots.

## Results

### Soft Substrates Do Not Impair Microglial Viability but Affect Their Morphology

**Figure 1:**
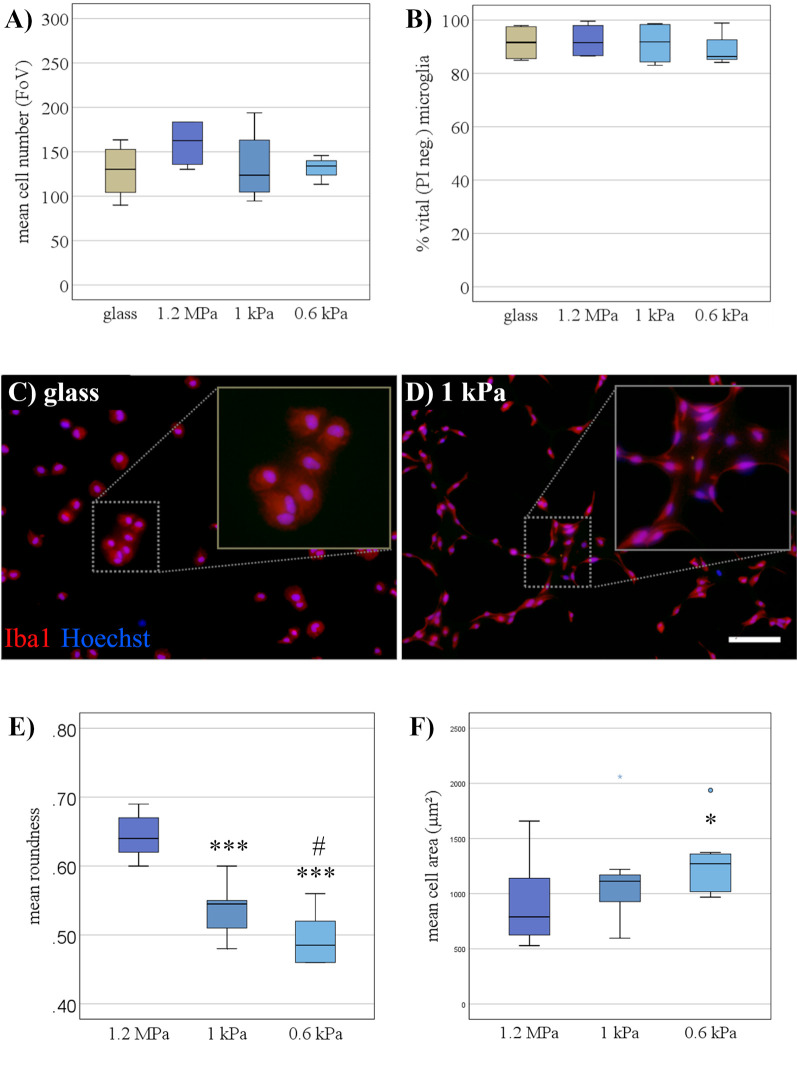
Soft substrates do not impair microglial viability, but affect their morphology. **(A)** When cultivated for 24 h, microglia showed no signs of increased cell death on PDMS substrates of any elasticity compared to a conventional glass surface, as monitored by the live-dead assay (*n* = 5). **(B)** Microglia numbers were not affected by the elasticity of PDMS substrates (*n* = 5). **(C)** Representative immunocytochemical images show microglia stained for “ionized calcium-binding adapter molecule 1” (Iba-1; red) and counterstained for nuclear marker Hoechst 33342 (blue) on conventional glass substrates and **(D)** PDMS-substrates of 1 kPa, mimicking the elasticity level of the living brain (scale bar = 100 μm). **(E)** Roundness (see “Materials and Methods” section for definition) of primary microglia decreased with the softness of the substrate (***p < 0.001 as compared to 1.2 MPa; ^#^*p* < 0.05 as compared with 1 kPa, *n* = 10). **(F)** The spread area of microglia expanded on softer substrates (*p < 0.05 as compared to 1.2 MPa; *n* = 10).

First, the viability of primary microglia grown for 24 h on soft PDMS-based substrates (1 kPa, < 1 kPa) was assessed in comparison to substrates of higher stiffness (1.2 MPa). There was no evidence of impaired cell viability as assessed by propidium iodide positive dead cells (Figure 1A) or overall cell number (Figure 1B), suggesting the PDMS substrates to be a suitable culture system for *in vitro* characterization of microglia. The purity of cultures was assessed by Iba-1 staining, yielding more than 90% Iba-1 positive microglial cells irrespective of substrate condition (Figures 1C,D). Furthermore, cell morphology of resting microglia was altered when cultured on softer substrates, predominantly displaying an elongated shape (Figure 1D). This was quantified as a significant decrease in cell roundness over the stiffness gradient (*F*_(2,27)_ = 48.8, *p* < 0.001, Ω^2^ = 0.88; Figure 1E) with significant differences between both softer substrates compared to the stiffer PDMS-control, as well as even between the softest conditions (1 vs. < 1 kPa; Figure 1E). Stainings confirmed that morphologically different microglia still expressed both the characteristic constitutive microglial marker CD11b as well as Iba-1 as a marker for activated microglia, while they did not express GFAP, indicating that there was no contamination with astrocytes (Supplementary Figure 1). Additionally, the mean cell spread area was significantly altered by substrate elasticity (*H*_2_ = 6.9, *p* < 0.05), with a significant increase by about 29% on the softest substrates (*M* = 1,260 μm^2^, SD = 90 μm^2^) compared to stiffer controls of 1.2 MPa (*M* = 900 μm^2^, SD = 110 μm^2^, *p* < 0.05, *r* = 0.83; Figure 1F).

### Increased Microglia Proliferation on Soft Substrates Under Resting Conditions

**Figure 2:**
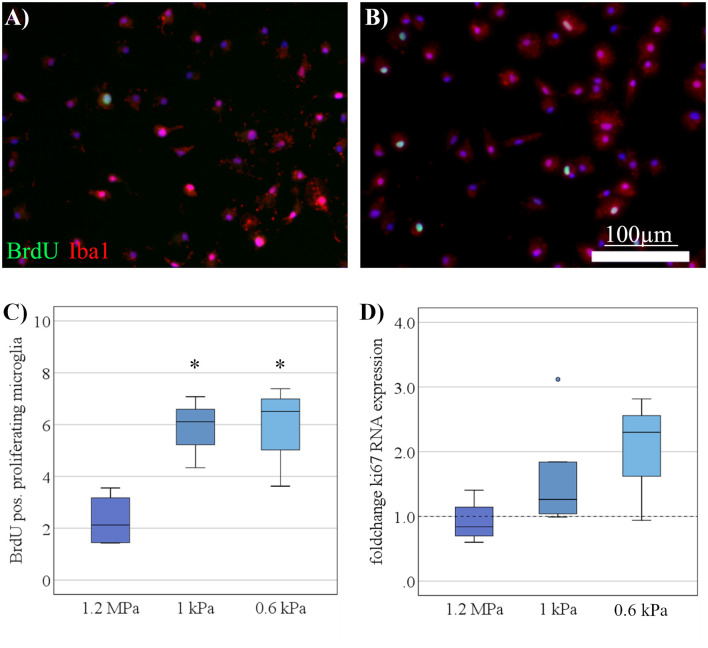
Increased microglia proliferation on soft substrates under resting conditions. **(A)** Representative images show Iba-1 positive microglia (red) double-stained for BrdU (green) to detect proliferating cells on substrates of 1.2 MPa, and **(B)** soft substrates of 0.6 kPa (scale bars = 100 μm). **(C)** Microglia proliferation measured by bromodeoxyuridine- (BrdU-) incorporation over 6 h increased with substrate elasticity (*p < 0.05 as compared to 1.2 MPa; *n* = 7). **(D)** In parallel, changes in microglia mRNA expression of Ki67 yielded an increasing yet statistically not significant trend on softer substrates (values displayed as fold change normalized to RPL13a and glass control; *n* = 5).

As a next step, the proliferative capacity of microglia was assessed when cultured on substrates of varying stiffness. Proliferative capacity increased on softer substrates as evaluated by BrdU uptake over 6 h (Figures 2A,B). The increase in proliferation on softer substrates was statistically significant (*F*_(2,12)_ = 8.8, *p* < 0.05, Ω^2^ = 0.4) with *post hoc* analyses revealing differences between substrates of 1 kPa (*M* = 5.8%, SD = 0.8%; *p* < 0.05) and the softest condition of <1 kPa (*M* = 6%, SD = 0.8%; *p* < 0.05; Figure 2C), both compared to stiffer controls (*M* = 2.3%, SD = 0.5%). We found a likewise, albeit non-significant, increase in ki67 mRNA expression on softer substrates (Figure 2D).

### Elasticity Affects Microglia Polarization Under Resting Conditions

**Figure 3:**
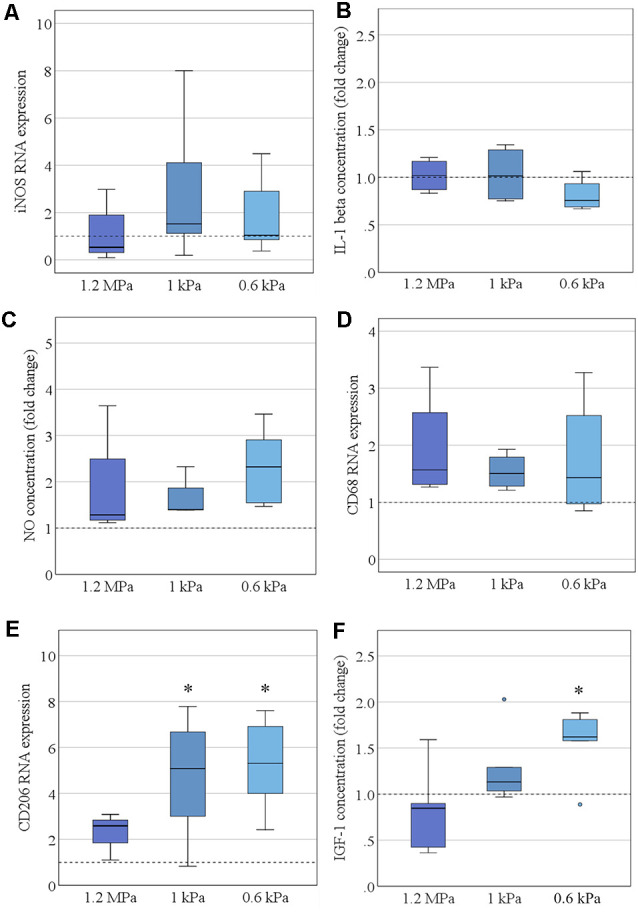
Elasticity affects microglia polarization under resting conditions. **(A)** Microglia RNA expression of the pro-inflammatory marker inducible nitric oxide synthase (iNOS) was not altered by substrate elasticity, when cultivated over 24 h (values displayed as fold change normalized to RPL13a and glass control, *n* = 9). **(B)** Correspondingly, the concentration of secreted pro-inflammatory cytokine interleukin-1β (IL-1 β) in the cell medium was not altered as quantified by ELISA (values displayed as fold change compared to glass control, *n* = 6). **(C)** Additionally, NO secretion was not altered in the cell culture medium as quantified by Griess Assay (values display as fold change compared to glass control, *n* = 6). **(D)** CD68 RNA expression revealed no change in the phagocytic activity of microglia (values displayed as fold change normalized to RPL13a and glass control; *n* = 6). **(E)** By contrast, the anti-inflammatory marker CD206 was significantly upregulated on RNA level when cultured on soft substrates (values displayed as fold change normalized to RPL13a and glass control, *p < 0.05 as compared to 1.2 MPa, *n* = 8). **(F)** In parallel, there was a likewise significant increase in anti-inflammatory insulin-like growth factor 1 (IGF-1) secretion within the cell medium (values displayed as fold change compared to glass control; *p < 0.05 as compared to 1.2 MPa, *n* = 6).

Consecutively, we assessed the spontaneous functional alteration of primary microglia cultured on soft substrates. Representative markers of pro- or anti-inflammatory microglia polarization states were assessed on the mRNA—as well as on the protein level. There was no pro-inflammatory alteration concerning varying stiffness levels, as assessed neither by iNOS mRNA expression (Figure 3A) nor by secretion of interleukin-1β (Figure 3B). Likewise, the secretion of NO into the medium was not altered on softer substrates (Figure 3C). There was no evidence of an increase in the phagocytic activity of microglia as evaluated by CD68 mRNA expression (Figure 3D). However, there was a significant increase in CD206 mRNA expression in an elasticity-dependent manner (*F*_(2,18)_ = 4.9, *p* < 0.05, Ω^2^ = 0.29; Figure 3E). We saw a more than 2-fold increase in CD206 levels on the softest substrates (*M* = 5.3, SD = 0.95) compared to stiffer controls of 1.2 MPa (*M* = 2.3, SD = 0.24). In parallel, we found a likewise significant increase in IGF-1 secretion within the cell medium (*H*_2_ = 7.6, *p* = 0.02, *r* = 0.78), with *post hoc* analyses revealing an almost two-fold increase on the softest substrates (*M* = 1.6, SD = 0.14, *p* = 0.02), compared to substrates of 1.2 MPa (*M* = 0.8, SD = 0.2; Figure 3F).

### Activation Marker Vimentin Is Differentially Expressed Dependent on Substrate Elasticity

**Figure 4:**
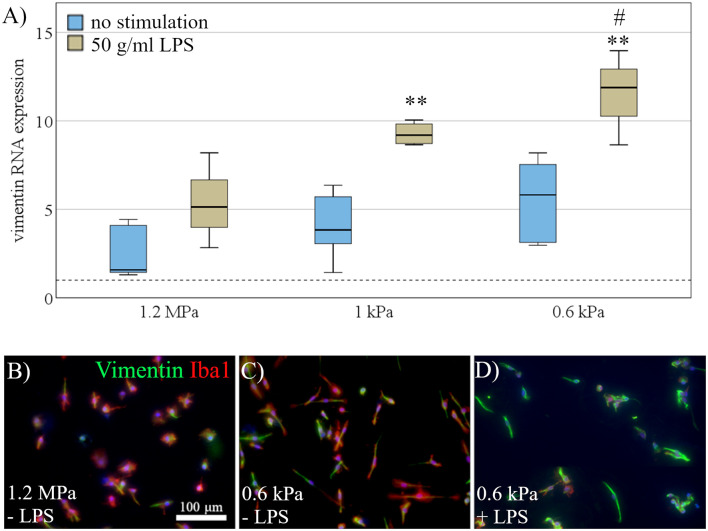
Activation marker vimentin is differentially expressed dependent on substrate elasticity. **(A)** Vimentin RNA expression was evaluated under rest (blue) and after stimulation with 50 mg/ml LPS (beige). Microglia exposed to LPS for 24 h upregulated vimentin compared to unstimulated cells. Additionally, two-way ANOVA revealed an additional effect of substrate softness independent of LPS, with an increase of vimentin expression on softer substrates (values displayed as fold change normalized to RPL13a and glass control; (**p < 0.01 as compared to 1.2 MPa, ^#^*p* < 0.05 as compared to 1 kPa; *n* = 3). **(B)** Representative images show Iba-1 positive microglia (red) co-stained for vimentin (green) and counterstained for nuclear marker Hoechst 33342 (blue) on substrates of 1.2 MPa, **(C)** soft substrates of 0.6 kPa, and **(D)** soft substrates of 0.6 kPa with additional stimulation of 50 mg/ml LPS (scale bar = 100 mm), showing an increase in vimentin expression on softer substrates, enhanced by LPS stimulation.

To further assess the effects of elasticity on microglial activation, we quantified vimentin expression as a marker of microglial activation. Vimentin RNA expression was evaluated both under resting conditions and after stimulation with LPS (as a strong pro-inflammatory stimulus). Data were fed into a two-way ANOVA to test for the interaction effect of stimulation and substrate stiffness, as well as both effects independently (Figure 4A). As expected, there was a significant effect of LPS stimulation on vimentin expression (*F*_(1,21)_ = 33.6, *p* < 0.001, Ω^2^ = 0.42; Figure 4A). Most interestingly, while we found no significant interaction effect, there was an additional effect of substrate stiffness independent of LPS stimulation (*F*_(2,21)_ = 10.1, *p* < 0.01, Ω^2^ = 0.25; Figure 4A). Further analyses for within-group effects revealed a significant increase in vimentin expression on LPS treated cells between the softest substrates (*M* = 11.5, SD = 2.7) compared to substrates of 1.2 MPa (*M* = 5.4, SD = 2.6, *p* < 0.01), while there was a likewise non-significant trend between the same groups in the unstimulated group (Figure 4A). Additionally, microglia were stained immunocytochemically for vimentin to detect vimentin on the protein level. Qualitatively—and corresponding to mRNA data—there was an increase in vimentin expression on softer substrates, additionally boosted by LPS stimulation, as shown by vimentin staining of Iba-1 positive microglia (Figures 4B–D).

### Dynamic Microglia Polarization Is Independent of Substrate Elasticity

**Figure 5:**
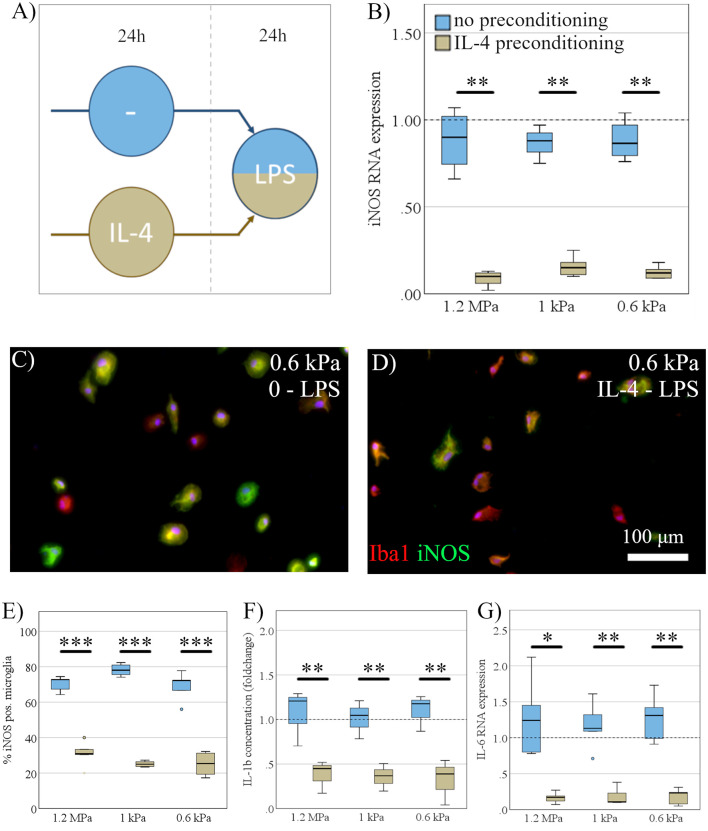
Dynamic microglia polarization is independent of substrate elasticity. **(A)** Microglia, seeded on substrates of varying stiffness, were exposed to LPS for 24 h, while either receiving an anti-inflammatory preconditioning stimulus (IL-4) for 24 h (beige) or no preconditioning (blue). **(B)** While IL-4 preconditioning exerted a robust inhibitory effect, there was no significant effect of substrate elasticity, as assessed by iNOS mRNA expression (*n* = 5). **(C)** Representative immunocytochemical images show Iba-1 positive microglia (red) counterstained for inducible nitric oxide synthase (iNOS; green), with nuclei stained blue on substrates of 0.6 kPa without preconditioning, and **(D)** IL-4 preconditioned cells (scale bar = 100 mm). **(E)** Expression of iNOS on protein level assessed immunocytochemically (*n* = 3) yielded the same results as on the mRNA level. **(F)** Parallel to iNOS, the concentration of secreted pro-inflammatory cytokine interleukin-1b (IL-1b) within the cell medium was not affected by substrate stiffness but was downregulated significantly by the anti-inflammatory stimulus, ELISA (*n* = 6). **(G)** Likewise, IL-6 RNA expression was significantly downregulated under an anti-inflammatory preconditioning stimulus without elasticity specific changes (*p < 0.05; **p < 0.01, ***p < 0.001; *n* = 6).

Next, we investigated the effect of substrate elasticity on dynamic polarization properties of microglia. To this end, microglia, seeded on substrates of varying stiffness, were stimulated with LPS for 24 h to induce a pro-inflammatory activation phenotype. Before LPS stimulation, microglia received either an anti-inflammatory preconditioning stimulus (IL-4) for 24 h, or no preconditioning stimulus, respectively (Figure 5A). Markers of pro-inflammatory microglia polarization were subsequently assessed on RNA and protein level. Anti-inflammatory pre-stimulation led to a significant reduction of pro-inflammatory markers upon LPS, constituting a microglia memory (Vay et al., 2018). In detail, a preceding exposure to IL-4 mitigated the pro-inflammatory response of microglia in terms of iNOS RNA expression (mean fold change no pro-conditioning = 1.2, SD = 0.6; mean IL-4 = 0.12, SD = 0.05; *F*_(1,29)_ = 46.9, *p* < 0.001, Ω^2^ = 0.62; Figure 5B), iNOS staining (mean fold change no pro-conditioning = 72.5% iNOS positiv microglia, SD = 6.8%; mean IL-4 = 27.1%, SD = 6.1%; *F*_(1,29)_ = 466.8, *p* < 0.001, Ω^2^ = 0.93; Figures 5C–E), IL-1β secretion (mean fold change no pro-conditioning = 1.3, SD = 0.5; mean IL-4 = 0.5, SD = 0.3; *F*_(1,17)_ = 43.2, *p* < 0.001, Ω^2^ = 0.75; Figure 5F), and IL-6 RNA expression (mean fold change no pro-conditioning = 1.4, SD = 0.9; mean IL-4 = 0.2, SD = 0.12; *F*_(1,29)_ = 30.9, *p* < 0.001, Ω^2^ = 0.5; Figure 5G). However, there was no specific effect of elasticity on their activation parameters in the context of “microglia memory.”

### Blockage of Chloride Channels Reverses Elasticity Dependent Effects

**Figure 6:**
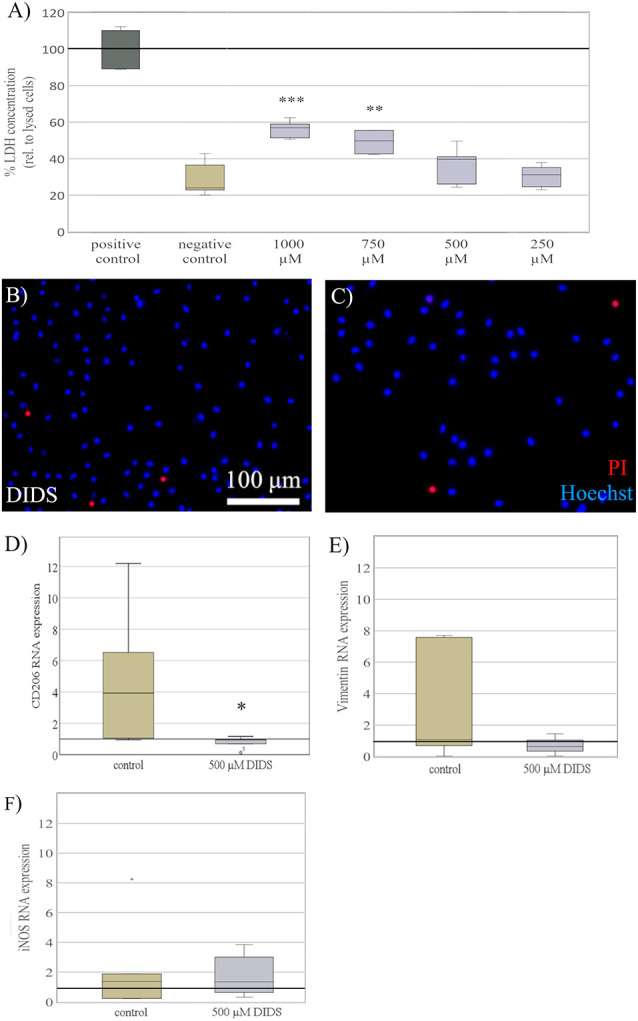
Blockage of chloride channels reverses elasticity dependent effects. **(A)** Microglia cultured on soft substrates of 0.6 kPa were treated with 4,4′-diisothiocyano-2,2′-stilbenedisulfonic acid (DIDS), to block stretch-activated Cl^−^ channels, for 24 h. Dependent on dosage, treatment with DIDS exerted toxic effects on microglia, as shown by LDH concentration normalized to the maximal LDH release of lysed cells as a positive control. A concentration of 500 μM DIDS revealed no increase in LDH release and was chosen for all further experiments. Media of cells treated with the solvent KHCO3 served as a negative control (values displayed as the mean percentage of LDH concentration relative to lysed; **p < 0.01 and ***p < 0.001 compared to negative control, *n* = 6). **(B)** Representative images of microglia stained for propidium iodide (red) counterstained with Hoechst 33342 (blue) show no increase in cell death in microglia treated with 500 μM of DIDS. **(C)** compared to KHCO_3_ controls. **(D)** After 24 h of exposure, blockage with DIDS showed a significant reversal of CD206 RNA expression on substrates of 0.6 kPa compared to controls treated with the solvent KHCO_3_ (*p < 0.05 compared to control, *n* = 5). **(E)** On the same note, blockage with DIDS revealed a non-significant trend in a reversal of vimentin RNA expression on substrates of 0.6 kPa (*n* = 4), **(F)** while the expression of iNOS RNA was not altered (*n* = 4).

To explore a potential mechanism behind some of the elasticity-evoked effects on microglia, we blocked stretch-dependent chloride-channels, previously shown to mediate microglial activation (Eder et al., 1998; Hines et al., 2009). Primary microglia were seeded on substrates of 0.6 kPa and treated with DIDS to block chloride channels. In order to establish a dosing regime, various concentrations of DIDS ranging from 250 μM to 1 mM were tested. There were dose-dependent signs of cell impairment under high concentrations of DIDS, as indicated by a significant increase in LDH release (*H*_4_ = 31, *p* < 0.001; Figure 6A), with a significant increase in LDH when treated with concentrations of 1 mM (*M* = 56% of positive control, SD = 7.9%; *p* < 0.001) and 750 μM (*M* = 45%, SD = 6.9%; *p* < 0.01), compared to negative controls treated with KHCO_3_ (*M* = 29%, SD = 7.9%). As a concentration of 500 μM DIDS did not negatively affect microglia as shown by both LDH release assay (Figure 6A) and propidium iodide staining (Figures 6B,C), this concentration was chosen to block chloride channels for further experiments. After 24 h of cultivation, blockage with DIDS completely abolished CD206 RNA expression induced in microglia by soft substrates 0.6kPa (*M* = 0.8, SD = 0.4) compared to control (*M* = 4.9, SD = 4.1; *t*_(8)_ = −2.5, *p* < 0.05, *d* = 0.89; Figure 6D). Furthermore, we saw a likewise, yet non-significant, trend on vimentin expression (control: *M* = 3.3, SD = 1.3; DIDS: *M* = 0.45, SD = 0.14; *t*_(11)_ = 4.2, *p* = 0.07; Figure 6E), while no difference was observed on iNOS expression (control: *M* = 2.1, SD = 2.5; DIDS = 1.7; SD = 1.2; *t*_(11)_ = 0.1, *p* = 0.7; Figure 6F).

## Discussion

While previously shown to affect various cell entities, like cardiomyocytes, neural stem cells, and neurons, the effects of elasticity of the microenvironment on microglia function remain elusive. As relevant viscoelastic alteration of neural tissue is induced by chronic inflammation (Streitberger et al., 2012; Millward et al., 2015), stroke (Freimann et al., 2013), or aging (Arbogast et al., 1997; Arani et al., 2015), further knowledge about elasticity-dependent microglia function will help to understand microglia action in disease better and possibly support directed therapeutic strategies.

To this end, we present evidence that substrate elasticity alters microglia morphology and substantially influences functional properties and activation markers of primary microglia *in vitro*. Importantly, the soft substrates resemble much more the elasticity of brain tissue than glass or plastic plates commonly used to culture microglia. Hence, the use of soft substrates may help to establish a more physiological environment in microglia culture experiments.

Our results indicate that microglia are converted to CD206 expressing microglia—one of the markers associated with M2-like microglia—when cultured on soft substrates mimicking the brain’s elasticity. Additionally, these changes are accompanied by a significant increase in proliferative activity, while overall microglial activation is enhanced as indicated by vimentin expression. In line with our results, Bollmann et al. (2015) reported that microglial morphology is affected by the microenvironment’s stiffness. Using polyacrylamide (PAA) substrates, they described a more complex microglia morphology displaying predominantly elongated shapes on softer substrates (Bollmann et al., 2015). While we here used cell roundness as a more general metric to quantify cell complexity, we found similar changes within the upper range of brain elasticity around 1 kPa. As known from the classical descriptions, microglial activation goes along with changes in cell morphology (Streit et al., 1988; Stoll et al., 1998). A highly arborized shape is associated with a resting microglia state while rounded stellate or even amoeboid shapes characterize activated microglia (Kreutzberg, 1996). Altogether, we suggest that microglia on soft substrates display essential properties of de-activated or resting microglia, more than microglia on hard substrates.

At the functional level, a previous report by Moshayedi et al. (2014) describes an increase in inflammatory microglia markers when cultured on stiffer substrates, as assessed by DNA microarray. Alike, we here observed an increase in anti-inflammatory markers on softer substrates, while we did not detect an alteration in pro-inflammatory markers. Additionally, converging with previous studies connecting anti-inflammatory states with microglial proliferation (Pepe et al., 2017; Vay et al., 2018), we found soft substrates to increase cell proliferation significantly.

Moreover, we observed a robust increase in vimentin expression by microglia cultured on softer substrates. Previous reports suggested an indispensable role of vimentin for initiating microglial activation (Jiang et al., 2012), as a marker of cell activation after axotomy (Graeber et al., 1988) or spinal cord injury (Noristani et al., 2017). Based on the importance of vimentin for microglial activation, the current data suggest a relevant impact of substrate elasticity on microglial activation, besides the predicted increase in vimentin expression upon LPS stimulation.

To sum up the functional aspects, we suggest that microglia on soft substrates are primarily resting, but more in a ready-to respond state than glass-plated microglia, as indicated by the increased proliferation capacity, mannose receptor expression (CD206), and vimentin expression. Within health and disease, microglia exert both pro-inflammatory (rather neurotoxic) or anti-inflammatory (rather neuroprotective) properties dependent on the given state of activation and polarization, all within a continuous and dynamically changing spectrum (Vay et al., 2018). Accordingly, microglia exhibit overlapping characteristic pro-inflammatory (“M1-like”) or anti-inflammatory (“M2-like”) markers, while “resting” microglia appear to display a more M2-like phenotype (Franco and Fernández-Suárez, 2015; Vay et al., 2018), which is in line with our findings. Moreover, tight temporal regulation and termination of microglia activation are vital, to ensure an adequate disease-state specific microglia reaction, i.e., after stroke (Hu et al., 2012; Walter et al., 2015), traumatic brain injury (Ramlackhansingh et al., 2011) or neurodegenerative disease (Hickman et al., 2018).

In the aging and diseased CNS, the stiffness of CNS tissue often decreases (Hiscox et al., 2018). Our results are compatible with the notion that those changes of mechanobiological properties may contribute to an altered functional and activation state of microglia, including a chronically activated phenotype, and a failure to shut down activation as known from neurodegenerative disorders (for recent reviews see Sabogal-Guáqueta et al., 2020; Webers et al., 2020).

Regarding the mechanism through which cells sense mechanical stimuli, previous studies highlighted the effect of stretch-dependent chloride channels by blockage with DIDS (Eder et al., 1998; Hines et al., 2009). In line with these results, the treatment of microglia on soft substrates with DIDS reversed the elasticity-induced expression of CD206 as a marker for M2-like microglia. Overall, while this highlights the robustness of our findings, the evidence is insufficient to determine stretch-dependent chloride channels as a causal substrate of all the observed effects. Moreover, DIDS might have additional non-specific effects, so future investigations should include NPPB and Flufenamic acid to make sure that the effects are indeed stretch-activated Cl^−^ channel-dependent and not anion-exchanger dependent (Eder et al., 1998; Schlichter et al., 2011). Thus, further investigations, i.e., by the usage of alternative pharmacological or molecular methods, are warranted to comprehensively clarify the mechanisms underlying elasticity-dependent effects on microglia. However, the strength of the current study is to characterize for the first time the effects of an abundant mechanical cue, i.e., elasticity, on microglia function, and suggest a potential mechanism as a basis for future studies.

Besides the static functional properties of microglia, recent studies highlight the importance of dynamic functional microglia states (Hamzei Taj et al., 2016; Vay et al., 2018; Rabenstein et al., 2020). In this respect, preconditioning of microglia, either pharmacologically or by a pathological stimulus, can modulate the successive microglia reaction upon further stimuli. As cultivation on soft substrates primed microglia towards an anti-inflammatory state under resting conditions, we hypothesized that this change of the starting position might also affect the dynamic reaction of microglia, or their “memory” (Vay et al., 2018). However, using established models of pro- or anti-inflammatory microglia stimulation, we found no significant effect of substrate elasticity on this microglia memory. Technically, our results reproduced previous reports that anti-inflammatory preconditioning with IL-4 alters the response upon a successive pro-inflammatory stimulation with LPS (Michelucci et al., 2009; Vay et al., 2018). Overall, while the effect of pharmacological stimuli far exceeded the rather moderate effect of substrate elasticity, we assume that potential effects on microglia dynamics in this study might have been obscured and could be provoked with less prominent pro-inflammatory stimuli.

Altogether, our data highlight that primary microglia is significantly affected by its microenvironment’s mechanical properties. Culturing microglia on substrates mimicking the softness of the living brain alters basal microglial activation levels. Thus, substrate elasticity appears to be relevant for the *a priori* configuration of microglia and may help to explain differences between histological examinations *ex vivo* and cultivation *in vitro*, important for translational research. Altogether, mechanical factors in general and elasticity, in particular, constitute an essential factor impacting how microglia interact with the CNS milieu and warrant further investigation.

## Data Availability Statement

The raw/processed data required to reproduce these findings cannot be shared at this time as the data also form part of an ongoing study.

## Author Contributions

SB, SD, AK, and DO conducted the experiments. J-AA, CH, MH, and NH prepared and calibrated the PDMS substrates. SB, SV, and MR performed the analysis and statistics. SB and SD drafted the manuscript. RM, BH, SV, MS, and MAR participated in the design and coordination of the study and critically revised to draft the manuscript. All authors contributed to the article and approved the submitted version.

## Conflict of Interest

The authors declare that the research was conducted in the absence of any commercial or financial relationships that could be construed as a potential conflict of interest.

## Supplementary Material

SUPPLEMENTARY FIGURE 1 **(A)** Representative images of microglia cultivated on soft PDMS substrates of 0.6 kPa expressing CD11b (red) as well as Iba-1 (green) as characteristic microglia markers. **(B)** Cultivation on soft substrates of 0.6 kPa did not promote contamination with primary astrocytes as indicated by staining for GFAP (red), but exclusively yielded growth of primary Iba-1 positive (green) microglia.
