# Supplementary material for: Substrate Elasticity Exerts Functional Effects on Primary Microglia
Source: Front Cell Neurosci. 2020 Nov 5;14:590500. doi: 10.3389/fncel.2020.590500 (PMC7674555; doi:10.3389/fncel.2020.590500)
Supplement: SUPPLEMENTARY FIGURE 1 — (A) Representative images of microglia cultivated on soft PDMS substrates of 0.6 kPa expressing CD11b (red) as well as Iba-1 (green) as characteristic microglia markers. (B) Cultivation on soft substrates of 0.6 kPa did not promote contamination with primary astrocytes as indicated by staining for GFAP (red), but exclusively yielded growth of primary Iba-1 positive (green) microglia. [file Image_1.pdf]

Suppl. Figure

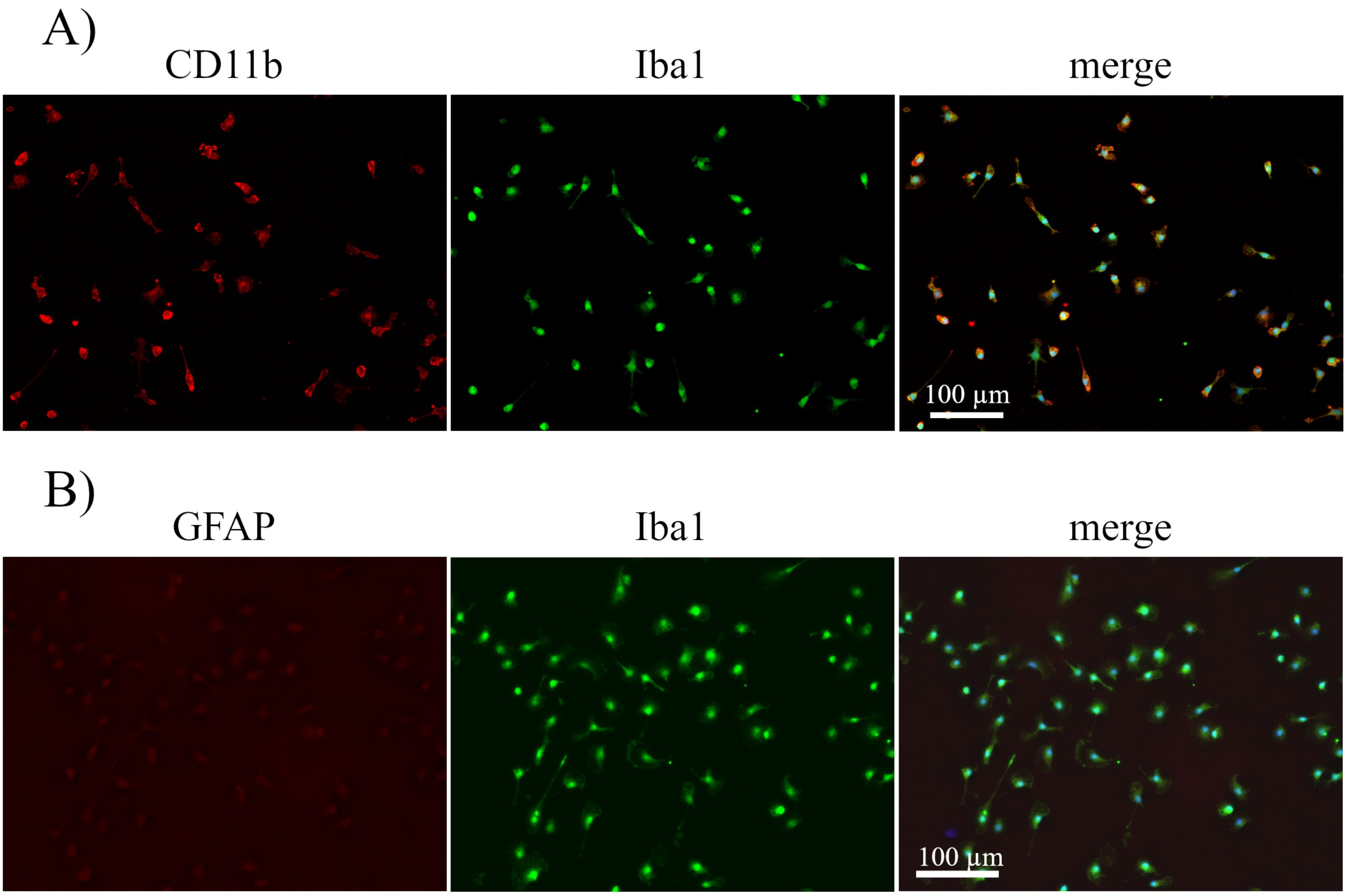

Suppl. Figure:

(A) Representative images of microglia cultivated on soft PDMS substrates of 0.6 kPa expressing CD11b (red) as well as Iba1 (green) as characteristic microglia markers.

(B) Cultivation on soft substrates of 0.6 kPa did not promote contamination with primary astrocytes as indicated by staining for GFAP (red), but exclusively yielded growth of primary Iba-1 positive (green) microglia.
